# Use of preoperative breast MRI to determine disease extent

**DOI:** 10.1186/bcr3769

**Published:** 2015-11-05

**Authors:** Nicky Dineen, Jennifer Weeks, Ruxandra Pietrosanu, Karina Cox, Pippa Mills

**Affiliations:** 1 Maidstone General Hospital, Maidstone, Kent, UK

## Introduction

Breast MRI can be performed in the preoperative workup of patients with biopsy-proven breast cancer to size lesions, if there is discrepancy regarding the extent of disease from clinical, mammography or ultrasound assessment, and to identify multicentric or multifocal disease. The purpose of breast MRI is to plan the optimum surgical procedure, thus reducing the local tumour recurrence rate and the need for the patient to undergo additional surgery.

## Methods

In this poster we have reviewed breast MRI examinations from patients with a new diagnosis of breast cancer, who had a preoperative MRI.

## Results

There were 75 scans in total. Patients who had MRI occult disease or neoadjuvant chemotherapy were excluded, leaving a total of 51 breast MRI scans. Invasive tumour size and total tumour size (invasive tumour + DCIS) as seen on MRI were compared with the size reported in the surgical pathology specimen. There was accurate correlation in invasive tumour size in 81% and significantly discordant sizing in 19%. Correlation in overall tumour size including DCIS was 70% and significantly discordant in 30%. In three patients in whom the total tumour size was overestimated, the patients consequently had complete local excision with wide excision margins. In another patient, in whom the total disease extent was underestimated on MRI, following complete local excision, repeat surgery was required for positive margins. In these four patients the MRI was misleading for guiding the optimum surgical procedure.

## Conclusion

MRI tumour size assessment particularly for DCIS should be interpreted with caution.

